# A rare case of type 1 leprosy reactions following tetanus infection in a borderline tuberculoid leprosy patient and a literature review

**DOI:** 10.1186/s40249-018-0441-4

**Published:** 2018-06-17

**Authors:** Chao Shi, Zhi-Chun Jing, De-Gang Yang, Jian-Yu Zhu

**Affiliations:** 1 Department of Leprosy, Shanghai Dermatology Hospital, No.1278 Bao De Road, 200443 Shanghai, People’s Republic of China; 2 Department of Therapy, Shanghai Dermatology Hospital, No.1278 Bao De Road, 200443 Shanghai, People’s Republic of China

**Keywords:** Type 1 leprosy reactions, Tetanus, Trigger

## Abstract

**Background:**

Type 1 leprosy reaction, also known as “reversal reaction”, is related to cellular immune responses to *Mycobacterium leprae* antigens. The risk factors that trigger type 1 leprosy reactions are poorly understood. Leprosy with concurrent tetanus is rare, and there are no publicly available reports of a leprosy patient infected with tetanus that induced type 1 leprosy reactions.

**Case presentation:**

A 56-year-old Chinese Han female presented to our hospital with symptoms of erythematous plaques and pain over her left upper limb for 2 days and foreign object sensation in her throat for 3 days. The patient had a 6-year history of leprosy. Type 1 leprosy reactions were initially considered, followed by treatment with methylprednisolone. Two days later, the patient’s symptoms were aggravated, with neck muscle tension and difficulty in opening her mouth, and the erythematous plaques had spread over most of her left upper limb. After further careful examinations, we confirmed the diagnosis of tetanus with concurrent type 1 leprosy reactions. The patient was given anti-tetanus treatment for 12 days and anti-leprosy reaction treatment for 4 months; the diseases were eventually controlled.

**Conclusions:**

This report suggests that tetanus infection may be a trigger for type 1 leprosy reactions.

**Electronic supplementary material:**

The online version of this article (10.1186/s40249-018-0441-4) contains supplementary material, which is available to authorized users.

## Multilingual abstract

Please see Additional file 1 for translations of the abstract into the five official working languages of the United Nations.

## Background

Type 1 leprosy reaction (T1LR), also known as “reversal reaction”, is related to cellular immune responses to *Mycobacterium leprae* antigens and mainly occur in borderline tuberculoid leprosy (BT), mid-borderline leprosy (BB) and borderline lepromatous leprosy (BL) patients [1]. T1LR can occur before, during or after anti-leprosy treatment, is associated with the mental and physical suffering of patients, and may lead to deformity and disability. The events or conditions that trigger T1LR are poorly understood.

Leprosy with concurrent tetanus is rare, and there are no publicly available articles of a leprosy patient infected with tetanus that induced T1LR. Here, we report a case of T1LR following tetanus infection in a BT patient.

## Case presentation

A 56-year-old Chinese Han female presented to Shanghai Dermatology Hospital in 2016 with symptoms of erythematous plaques and pain over her left upper limb for 2 days and foreign object sensation in the throat when swallowing for 3 days. The patient had a 6-year history of leprosy. She was diagnosed with BB in 2011 and received multidrug therapy (MDT) (600 mg of rifampin and 300 mg of clofazimine monthly; 100 mg of dapsone and 50 mg of clofazimine daily) for 1 year, resulting in a clinical cure in 2012.

T1LR were initially considered, followed by treatment with 20 mg/day of methylprednisolone given orally. Two days later, the patient’s symptoms were aggravated, with neck muscle tension and difficulty in opening her mouth, and the erythematous plaques had spread over most of her left upper limb.

**Fig. 1:**
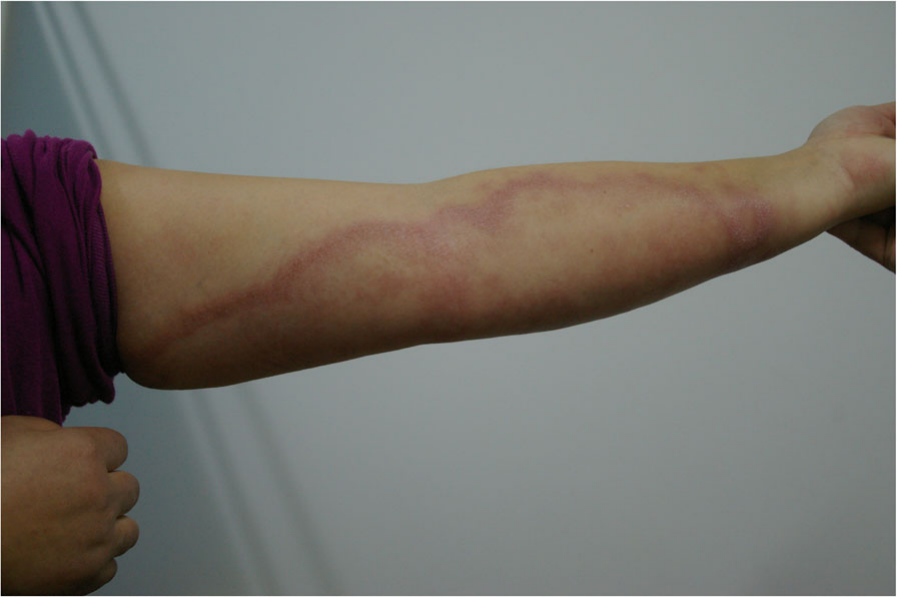
Erythema resulting from type 1 leprosy reactions. The patient’s left arm shows large erythematous plaques with infiltrated edges, sparing the central region

On physical examination, the patient had a normal blood pressure and pulse with a temperature of 37.8 °C, but she displayed shortness of breath. Her facial expressions included a wry smile and trismus, with the corners of her mouth pulling outward and upward, and she had difficulty speaking. The patient’s abdominal muscles were too stiff for palpation of the patient’s liver and spleen. Persistent stiffness was found in the neck and four limbs, together with opisthotonus and occasional paroxysmal spasms. Anesthetic erythematous plaques were observed over her left upper limb (Fig. 1). The bilateral ulnar nerves and right common peroneal nerve were thickened and exhibited tenderness. The patient exhibited right foot drop, atrophy of the extensor of the right lower leg, and an ulcer on the right foot. Laboratory examinations showed a white cell count of 12 000/mm^3^ (reference value: 3690–9160/mm^3^) and neutrophils 81% (reference value: 50–70%). Her liver and renal function tests were normal. Slit skin smears showed the presence of acid-fast bacilli ranging from negative to a score of 1+ at 6 different sites. According to her medical history and clinical symptoms, she was diagnosed with tetanus and BT accompanying T1LR.

This patient was given the following: 100 000 IU tetanus antitoxin in a 500-ml 5% glucose-saline intravenous infusion daily; 200 000 U penicillin in an intramuscular injection four times a day; and 200 mg of hydrocortisone in a 250-ml 5% glucose intravenous infusion daily. Additionally, 10 mg/day of diazepam and 50 mg/day of phenergan were given by intramuscular injection. The patient was hospitalized in a dark, quiet room to reduce light stimulation and prevent spasms. In addition, a dental pad was placed in the oral cavity to prevent the patient from biting her tongue. The ulcer on her right foot was debrided every day, followed by rinsing with 3% hydrogen peroxide and injection of 20 000 IU tetanus antitoxin around the wound. The paroxysmal spasticity and intensity of the spasms started to decrease after 5 days. Twelve days later, she was discharged from the hospital in stable condition. She was followed up in our outpatient department and treated with MDT and 40 mg/day of prednisone orally with taper to prevent T1LR for 4 months; the erythematous plaques and neuropathic pain eventually subsided.

## Discussion

*Clostridium tetani* is widely present in the natural environment; its spores can invade the body through damaged skin or mucosa, and it can reproduce locally, causing spasms and producing hemolytic exotoxin. Tetanus exotoxins are generally considered to spread centripetally along peripheral nerves to the corresponding region of the spinal cord and then enter central motor nerve bundles, leading to skeletal muscle spasms and convulsions [2]. In humans, the occurrence of tetanus after infection with *C. tetani* depends on the amount of exotoxin released, previous specific immunity, and the speed of establishment of an effective immune response [2, 3].

Leprosy patients often have superficial sensory disturbances in their extremities; they are also vulnerable to trauma and often have chronic ulcers after long-term treatment, which can lead to other anaerobic or aerobic secondary infections [4]. However, concurrent *C. tetani* infection is far less common in leprosy patients than it is in the general population. Tetanus in leprosy is rare and reported occasionally as case reports only [5, 6]. According to a survey performed by Smith, the incidence of death from tetanus in the general population is 4.98% in Karnataka Province, India, but none of the 67 leprosy patients died of tetanus [7]. In 1988, Hodes and Teferedegne first reported five cases of leprosy with concurrent tetanus in Ethiopia [8].

Saha et al. detected protective antibodies in a significantly higher proportion of non-immunized lepromatous leprosy patients compared to non-immunized matched healthy controls, suggesting that lepromatous leprosy patients are somewhat protected against tetanus [9]. Furthermore, Singh et al.’s findings indicate immunological protection from tetanus and support the clinical impression of the rarity of tetanus in leprosy patients [10]. Dastur et al. speculated that acquired immunity for tetanus may be due to stimulation of the immune system by repeated infection by *Clostridium*. Leprosy patients often have chronic ulcers with an accompanying high likelihood of bacterial contamination, which provides them with much stronger resistance to tetanus than found in the general population [11]. Although the mechanisms that protect leprosy patients from tetanus have not been fully elucidated, the above three studies indicate and propose at least one possibility [9–11].

T1LR are believed to be the result of the spontaneous enhancement of cellular immunity and delayed hypersensitivity to *M. leprae* antigens, though the causes and mechanisms of this enhancement remain unclear [12]. Previous studies have shown that the increasing risk of T1LR is due to several factors, such as intercurrent infections, stress, trauma, use of oral contraceptives, increasing age, and extensive disease [13–15]. Some researchers think intercurrent infections favors the intracellular cytokines expression and, probably, the inflammatory reaction operating as a stimulatory signal triggering the leprosy reactions [16, 17]. The leprosy patient in this study, who had achieved clinical cure for many years, showed symptoms of T1LR after a few days of tetanus infection, suggesting that cellular immunity activated by tetanus might have occurred.

T1LR present as the induration and erythema of existing lesions, frequently with prominent acral edema and often with progressive neuritis, causing sensory and motor neuropathy [12, 14]. Given their anti-inflammatory properties, oral and intralesional corticosteroids are typically highly effective for the clinical treatment of T1LR [18]. However, it remains controversial whether antibacterial therapy is necessary during the treatment of T1LR, with some researchers suggesting that antimicrobial therapy should be continued throughout treatment [12, 14]. Based on evidence of the presence of acid-fast bacilli in slit skin smears of the patient (1+) at 4 years after treatment cessation, we reintroduced MDT as antimicrobial treatment.

Leprosy reactions, particularly T1LR, are often accompanied by peripheral nerve damage, e.g., involvement of the 9th and 10th pairs of cranial nerves can present as dysphagia [19, 20]. However, the patient in this study reported symptoms and complaints of the sensation of a foreign object in her throat when swallowing, which affected her ability to eat. Hence, the symptoms of the patient were initially misdiagnosed during her first visit to our outpatient facility as a manifestation of a leprosy reaction. After further careful examination, we confirmed the diagnosis of tetanus with concurrent T1LR. T1LR involve no trismus, opisthotonus, muscle rigidity, or paroxysmal spasms. Thus, the two diseases should not be difficult to differentiate upon cautious diagnosis.

## Conclusion

This report first demonstrates a rare case of T1LR following tetanus infection, suggesting that tetanus infection may be a trigger for T1LR.

## Additional file

Additional file 1: Multilingual abstract in the five official working languages of the United Nations. (PDF 856 kb)

## Abbreviations

BB: Mid-borderline leprosy
BL: Borderline lepromatous leprosy
BT: Borderline tuberculoid leprosy
MDT: Multidrug therapy
T1LR: Type 1 leprosy reaction
