# Supplementary material for: A rare case of type 1 leprosy reactions following tetanus infection in a borderline tuberculoid leprosy patient and a literature review
Source: Infect Dis Poverty. 2018 Jun 17;7:58. doi: 10.1186/s40249-018-0441-4 (PMC6004287; doi:10.1186/s40249-018-0441-4)
Supplement: Supplementary file 1 — Multilingual abstract in the five official working languages of the United Nations. (PDF 856 kb) [file 40249_2018_441_MOESM1_ESM.pdf]

Translation of the abstract into the five official working languages of the United Nations

حالة نادرة لتفاعلات مرض الجذام من النوع الأول أعقبت الإصابة بعدوى الكزاز لدى مريض بالجذام شبه الدرني الحدي واستعرض ما كُتب عنه

قدمه: تشاو شي، وتشي تشون، ودي جانج يانج، وجيان يو تشو

الملخص
المعلومات الأساسية: إن تفاعل مرض الجذام من النوع الأول، والذي يُعرف كذلك باسم "التفاعل العكسي"، يرتبط باستجابات الخلايا المناعية لمولدات مضادات البكتيريا الفطرية الجذامية. إن عوامل الخطر التي تتسبب في الإصابة بتفاعلات مرض الجذام من النوع الأول غير مفهومة بدرجة واضحة. فحدوث مرض الجذام المتزامنة مع الإصابة بالكزاز أمرٌ نادر، وكما أنه ليس هناك تقارير متوفرة حول مريض بالجذام مصاب بعدوى الكزاز التي نجم عنها تفاعلات مرض الجذام من النوع الأول.
عرض الحالة: عُرضت علينا بالمستشفى حالة لامرأة صينية من عرق الهان تبلغُ من العمر ٥٦ عاماً تظهر عليها أعراض صفائح بقع حماموية وتعاني من ألم في أعلى ذراعها اليسرى لمدة يومين والشعور بجسم غريب في حنجرتها لمدة ٣ أيام. كان لدى المريضة تاريخ بالإصابة بمرض الجذام لمدة ٦ سنوات. كانت تعتبر في بادىء الأمر أنها مصابة بتفاعلات مرض الجذام من النوع الأول، وأعقب ذلك المعالجة عبر استخدام مركب الميثيل بريدنيزولون. فبعد مرور يومين، تفاقمت أعراض المريضة، مصحوبة بتوتر في عضلة العنق وصعوبة في فتح فمها، وقد انتشرت صفائح البقع الحماموية في معظم أعلى ذراعها اليسرى. وبعد دراسة الحالة على نحوٍ متأنٍ، تم تأكيد التشخيص بالإصابة بالكزاز المتزامن مع حدوث تفاعلات مرض الجذام من النوع الأول. تلقت المريضة علاج مضاداً للكزاز لمدة ١٢ يوماً وعلاجاً مضادًا لتفاعل مرض الجذام لمدة ٤ أشهر; وفي نهاية المطاف تمت السيطرة على الأمراض.
الاستنتاجات: يُشيرُ هذا التقرير إلى أن عدوى الكزاز قد تتسبب في حدوث تفاعلات مرض الجذام من النوع الأول.

Translated from English version into Arabic by Bashaier Allam, proofread by GHANIA Khalifa, through

TRANSLATORS WITHOUT BORDERS

一例罕见的界限类偏结核样型麻风患者感染破伤风后并发 1 型麻风反应的病例报道及相关文献综述

石超，景志春，杨德刚，朱建宇

摘要
引言：1 型麻风反应又称为“逆向反应”，属于机体对麻风杆菌抗原的细胞免疫反应。导致 1 型麻风反应发生的诱因目前还知之甚少。麻风患者并发破伤风感染十分罕见，而由破伤风感染诱导 1 型麻风反应发生的报道在现有文献中还未见记载。
病例介绍：56 岁中国汉族妇女，因左上肢红斑伴有疼痛 2 d，咽部吞咽时有异物感 3 d 来医院就诊。患者有 6 年麻风病史。首诊考虑患者病症为 1 型麻风反应，给予甲基强的松龙治疗。2 d 后患者症状加重，出现颈部肌肉强直和张口困难，以及左上臂红斑扩大。经进一步仔细检查后，确诊患者为破伤风感染并发 1 型麻风反应。经过 12 d 抗破伤风治疗和 4 个月抗麻风反应治疗后，患者病情最终得以控制。
结论：破伤风感染很可能是这例麻风患者 1 型麻风反应发生的诱因。

Translated from English version into Chinese by Jian-Yu Zhu

Description d’un cas rare de réaction lépreuse de type 1 consécutive à une infection tétanique chez une patiente atteinte de lèpre tuberculoïde dans un état limite et revue de la littérature

Chao Shi, Zhi-Chun Jing, De-Gang Yang, Jian-Yu Zhu

Résumé

Contexte: la réaction lépreuse de type 1, ou «réaction d'inversion», est associée aux réponses immunitaires cellulaires aux antigènes de *Mycobacterium leprae*. Les facteurs de risque à l'origine de cette réaction sont mal connus. La concomitance de la lèpre et du tétanos est rare et aucun rapport publié ne fait état d'un patient lépreux atteint simultanément d'un tétanos ayant provoqué une réaction lépreuse de type1.

Présentation du cas: une femme chinoise âgée de 56 ans, appartenant au groupe ethnique Han, s'est présentée à notre hôpital avec les symptômes suivants : plaques érythémateuses et douleurs à la surface du membre supérieur gauche depuis 2 jours et sensation de corps étranger dans la gorge depuis 3 jours. Cette patiente était porteuse de la lèpre depuis 6 ans. Le personnel médical a d'abord envisagé une réaction lépreuse de type 1 et mis en place un traitement à la méthylprednisolone. Deux jours plus tard, les symptômes de la patiente se sont aggravés avec l'apparition d'une tension musculaire dans la nuque, de difficultés à ouvrir la bouche et de plaques érythémateuses sur la majeure partie du membre supérieur gauche. Après de nouveaux examens approfondis, nous avons confirmé le diagnostic du tétanos associé à une réaction lépreuse de type 1 concomitante. La patiente a reçu un traitement antitétanique pendant 12 jours, complété par un traitement de la réaction lépreuse pendant 4 mois. Les deux maladies ont finalement été jugulées.

Conclusions: le présent rapport suggère qu'une infection tétanique peut être à l'origine d'une réaction lépreuse de type 1.

Translated from English version into French by Suzanne Assenat, proofread by Eric Ragu, through

TRANSLATORS WITHOUT BORDERS

Редкий случай лепрозной реакции 1 типа с последующим заболеванием столбняком у пациента с пограничной туберкулоидной лепрой и литературный обзор.

Чао Ши, Чжи-Чунь Цзин, Дэ-Ган Ян, Цзянь-Юй Чжу

Реферат

Справочная информация: Реакция лепры 1 типа, также называемая «реверсивной реакцией», относится к клеточным иммунным ответам на антигены *Mycobacterium leprae*. Не вполне понятны факторы риска, вызывающие реакции лепры 1 типа. Одновременное заболевание лепрой и столбняком происходит редко; при этом отсутствует публично доступная документация о пациентах, зараженных лепрой и столбняком, индуцировавшим лепрозную реакцию 1 типа.

Клинический случай: В нашу больницу была доставлена 56-летняя китайская женщина Хань с симптомами эритематозных бляшек, болью в левой верхней конечности, продолжавшейся последних 2 дня, и ощущением постороннего предмета в горле на протяжении 3-х дней. Данная пациентка имеет шестилетнюю историю заболевания лепрой. Первоначально были исследованы реакции лепры 1 типа, с последующим введением метилпреднизолона. Спустя два дня симптомы пациентки ухудшились, появилась напряженность мышц шеи и трудности с открытием рта, эритематозные бляшки распространились по большей части её левой верхней конечности. В результате тщательного обследования мы подтвердили диагноз столбняка с одновременными лепрозными реакциями 1 типа. Пациентка прошла 12-дневный курс лечения от столбняка и 4-месячный курс лечения от лепрозных реакций; в конечном счете, заболевания были взяты под контроль.

Заключение: Данный отчёт показывает, что инфекция столбняка может провоцировать лепрозные реакции 1 типа.

Translated from English version into Russian by Liudmila Tomanek, proofread by Ekaterina Rugg, through

Presentación de un caso raro de reacción de lepra tipo 1 posterior a una infección por tétanos en una paciente con lepra tuberculoide límite y revisión de la literatura

Autores: Chao Shi, Zhi-Chun Jing, De-Gang Yang y Jian-Yu Zhu

Resumen

Antecedentes: La reacción de lepra tipo 1, también conocida como "reacción reversa", está relacionada con respuestas celulares inmunes a *los antígenos de* Mycobacterium Leprae. Los factores de riesgo que desencadenan las reacciones de lepra tipo 1 no se comprenden muy bien. La lepra concurrente con tétanos es inusual, y no hay reportes publicados sobre pacientes de lepra infectados por tétanos que haya inducido una reacción de lepra tipo 1.

Presentación del caso: Una paciente china de la etnia Han de 56 años se presentó en nuestro hospital con síntomas de placas eritematosas, dolor en el miembro superior izquierdo y sensación de cuerpo extraño en la garganta de dos y tres días de evolución respectivamente. La paciente tenía antecedentes de lepra de seis años de evolución. Inicialmente consideramos una reacción de lepra tipo 1, que tratamos con metilprednisolona. Dos días después, los síntomas de la paciente se agravaron, con tensión de la musculatura del cuello y dificultad para abrir la boca. Las placas eritematosas se habían extendido por casi todo el miembro superior izquierdo. Tras un examen clínico más exhaustivo, confirmamos el diagnóstico de tétanos concurrente con una reacción de lepra tipo 1. La paciente fue sometida a tratamiento contra el tétanos por 12 días y a tratamiento contra la reacción leprosa por cuatro meses. Al final, logramos controlar las patologías.

Conclusiones: Este reporte sugiere que la infección por tétanos puede desencadenar reacciones de lepra tipo 1.

Translated from English version into Spanish by George Simon, proofread by Marta Callava Linares, through
